# A Local Survey on Nursing Practices for Infection Prevention in a Cardiac-surgery Intensive Care Unit

**DOI:** 10.37825/2239-9747.1056

**Published:** 2024-07-25

**Authors:** Vincenzo Andretta, Valentina Cerrone, Eros De Simone, Ornella Piazza

**Affiliations:** a Management Planning and Control Unit, Salerno University Hospital “San Giovanni di Dio e Ruggi D’Aragona”, Salerno, Italy; b Oncology Unit, Salerno University Hospital “San Giovanni di Dio e Ruggi D’Aragona”, Salerno, Italy; c Cardiac Surgery Intensive Care, Salerno University Hospital “San Giovanni di Dio e Ruggi D’Aragona”, Salerno, Italy

**Keywords:** Healthcare-associated infection, Infection prevention and control, Nursing, ICU

*Dear Editor*,

Infection Prevention and Control (IPC) are vital components of patient management in the Intensive Care Unit (ICU), particularly in high-risk environments like post Cardiac Surgical ICUs. Preventing and controlling infections in post-cardiac surgery ICUs require a comprehensive strategy that includes thorough hand hygiene, correct use of personal protective equipment (PPE), following aseptic techniques during invasive procedures, proper environmental cleaning, and antibiotic stewardship protocols [1]. Even with evidence-based guidelines being accessible, the application of these measures can differ greatly within healthcare settings because of factors like resource availability, staff adherence, and organizational culture.

We wanted to assess nurses, compliance with established protocols, identify strengths and areas for improvement in the post-cardiac surgery ICU of an Italian University Hospital. We interviewed 20 full-time nurses on two topics:

- Ventilator-associated pneumonia (VAP) precautions;
- Catheter-related bloodstream infection (CRBSI) precautions;

The questionnaires used in this study were developed by the authors specifically for this research and are not validated instruments. The development involved a detailed examination of current literature and guidelines, which included suggestions from the Centers for Disease Control and Prevention (CDC) and other pertinent sources [2–4].

About the activities carried out to prevent VAP, nurses follow about 80% of the measures reported as useful but, in the cardiac surgery ICU, they did not follow VAP prevention guideline when contrasting with more immediate need of hemodynamic stabilization, such as holding the patient with the head raised at 30–45° in the semi-reclining position. This is a practice carried out only if the patient’s clinical conditions allow it and, in most cases, patients are in semi-Fowler position. From the data analysis, there is no periodic monitoring of the endotracheal tube cuff pressure, while as regards the use of the sterile technique during airway suction, all nurses agree in the use of the no-touch technique.

As the CRSBI precautions is concerned, there is an adequate response from nursing care for most of the activities, but the ICU is not equipped with CVCs impregnated with chlorhexidine/silver sulfadiazine or minocycline/rifampicin when the catheter is expected to remain in place for more than 5 days. Needle-free intravascular catheter access systems are used.

Shortcomings like the absence of regular checks on endotracheal tube cuffs and the limited availability of specialized catheter options point to areas in need of enhancement and additional resource distribution. It is crucial to improve overall infection control measures by ensuring consistent documentation practices and addressing equipment shortages. The teamwork seen in this study reflects the diverse aspects of infection control efforts in ICUs. Effective communication and collaboration [5] are necessary for the smooth execution of infection prevention measures.

Improving these aspects can additionally boost patient safety and lessen healthcare-associated infections. Ongoing surveillance, instruction, and teamwork are crucial for upholding top-notch infection control in critical care environments.
